# Complicate Fillings

**Published:** 1870-10

**Authors:** M. S. Dean


### ARTICLE II.

### *Complicate Fillings.*

By M. S. Dean, D.D.S.

Read before the Illinois State Dental Society.

We are becoming daily more strongly convinced that many failures occur in our operations upon the teeth which might have been easily prevented had complicate fillings been resorted to instead of simple ones.

By complicate fillings we mean such fillings as combine two or more simple fillings in one.

In opening this subject for discussion, (for this is the chief object of this paper,) I shall attempt merely to direct attention to such cases in practice as seem to indicate complicate fillings, and offer some suggestions as to the preparation of such cavities and the proper mode of filling them.

In the first place, I will direct attention to the bicuspid teeth, for in these such fillings are more frequently required. We often find small cavities in the anterior and posterior pits connected by an almost imperceptible fissure, and sometimes instead of this fissure a roughened appearance of the enamel, threatening disintegration, though, as yet, showing no visible evidence of decay.

In the case with a slight fissure, I would open it to the depth of decay, or in case the depth is not sufficient to admit a strong band of union, cut a little beyond the depth of the decay; then, in my retaining pit in the anterior cavity fasten a strip of heavy foil, (say 30 or 60) and carry it over to the pit in the posterior cavity. By this means we may fill the fissure if there be no undercut, so securely that it cannot be removed without displacing the gold in the anterior and posterior cavities, and save, frequently, more of the strength of the tooth than we could otherwise do. When, instead of this fissure, we find roughened enamel, which we know must yield sooner or later to destroying agents, and which cannot be cut or polished out without removing too much of the tooth substance, we would treat the case as if decay had already commenced.

Again, take the tooth which we have just filled, and suppose there be in addition to this complicate cavity, one in the posterior approximal surface. Now, unless the enamel on the posterior ridge be very perfect, and unless the coronal wall of the cavity be so strong that it will not be liable to break down even by hard usage, I would, by all means, before filling the complicate cavity on the grinding surface, cut away the posterior ridge and unite them all in one; and this I would do if there was already room to approach the posterior approximal cavity. But, if there was not room, and the cavity a very large one, I should, even though the coronal wall appear sufficiently strong, drill through from the cavity in the posterior pit into this cavity; then with chisels cut away the margin and unite them all in one, thus making a direct opening into the postesior approximal cavity, which would greatly facilitate the operation, and render it *possible* to make a perfect filling at the point where the greater number of failures occur—the cervical margin.

If, again, there was an anterior approximal cavity in this same bicuspid, I would treat it (other things being equal,) the same as the posterior one, making of them all one complicate filling.

Let us again, for example, take this same tooth, and suppose it to have been left a year or two longer before it was filled. Now the buccal and palatal wall of the approximal cavities have lost their supporting dentine; decay has advanced beyond the margin of the gums and extended into the neck of the tooth, and is in dangerous proximity to the pulp, which is sensible to thermal changes. If we fill one of these approximal cavities singly, we must depend upon a small retaining point at the cervical wall, and upon the slight concavity of the enamel which forms the buccal and palatal walls, and perhaps, the overhanging posterior ridge, for we cannot drill other retaining pits without endangering the pulp. Should we succeed in filling such a cavity without fracturing the walls, the filling could not be of long duration, the walls would soon crumble away and the filling drop out. In such a cavity it might also be highly important to cover that portion nearest the pulp with some nonconducting material, which would render it still more difficult to secure the filling. In the above, and like cases, complicate fillings are most strongly indicated, which should extend from the anterior to the posterior approximated cavities. And although I should not destroy the symmetry of the tooth unnecessarily, I should remove the buccal and palatal walls until I found them sufficiently strong to promise durability, and drill my retaining pits horizontally or perpendicular, or both, from or near the grinding surface. One or more pits are absolutely necessary at the base of the cavity near the cervical margin. After this complicate cavity is prepared and ready for filling, we should commence with the posterior cervical pit, and fill this carefully with No. 30 foil—carefully, for a careless blow might split off the thin wall of this retaining pit, or the instrument misdirected might pass between the root and the rubber dam. Now, fearing this slight point might be broken or lossened by the repeated blows from the mallet, should we attempt to build up the filling upon it unsupported by others, it will be safer to fill the nearest pit or pits and join them together with heavy foil; then commence at the base of the cavity, using small pellets or small pieces of heavy foil, whichever can be manipulated the most readily, and with a small foot plugger with the point extending over and slightly beyond the cervical margin, build up the filling, but before it is finished the filling in the anterior approximal cavity should be so far advanced that they may be firmly bound together with strips of heavy foil. The work has now become simple and the mode obvious; and I will merely add that if either or both of the cusps are pointed and not very strong, I should consider the safety of the tooth better secured if they were filed down and covered with thick foil, as otherwise there would be great danger of their being split off in masticating hard substances.

Now, gentlemen, we will take for an example the first superior molar. We find cavities in the central and posterior pits, and a posterior approximal cavity. From the posterior pit a fissure leads over the palatal surface, extending to within a line of the gum and terminating in a small cavity. From this fissure on the palatal surface, one leads off, following the dividing seam which separates the anterior depressed cusp from the anterior palatal cusp.

Now, if the ridge which separates the central and posterior pits be not strong and perfect I would make one cavity of these. If the coronal wall of the posterior cavity be not already broken, I should remove that portion which divides the cavity in the posterior pit from the posterior approximal cavity. Then with a file suitable for that purpose open the palatal fissure; then with a drill, properly prepare the palatal cavity, and drill out the fissure that leads around the anterior depressed palatal cusp. In this latter work especially, we find Green's Pneumatic Engine invaluable. Having prepared this complicate cavity, the filling should be commenced in the retaining pit in the cervical margin of the approximal cavity, uniting it as soon as necessary or convenient with the cavity in the posterior pit, taking care not to clog up the avenue to the base of the filling, and then continue as detailed in filling the posterior approximal cavity of the bicuspid. Before this is completed, however, I should fasten a strip of heavy foil in the posterior cavity, and lead over to the cavity in the palatal surface. From the palatal cavity, with a strip of heavy foil, follow around the depressed cusp until I came to the terminus of the fissure, where I should fasten it in a properly shaped retaining pit. It will be needless to detail farther the mode of filling this complieate cavity. Such cavities are not uncomonly found in practice; indeed, during the past week I have filled eight of this character which would answer very well the description here given.

It will not be necessary for me to go into a detailed description of the complicate cavities in the lower bicuspids and molars, as the same priuciples apply to these, with a little variation in practice. There is, however, a certain class of under molar teeth where the enamel upon the entire grinding surface is found very imperfect when they are first erupted, of which I will say a word. Here the enamel is uneven or convoluted, as if imperfectly fused. If disintegration has not actually commenced in these, I would polish out the roughness; but if, as is commonly the case, we find a lingual and labial fissure, also small cavities already formed in different portions of the grinding surface, not well defined, but with the fissures branching off in various directions, I should cover the entire surface with a complicate filling. In some parts the complicate cavity would be of slight depth, in others deep enough to make good strong retaining points. This class of teeth is by no means rarely to be found, at least in large cities.

I will say further in regard to the first lower molars, that they are about the worst abused teeth in the whole dental family. We often find one of the pits on the grinding surface filled with a little round plug of gold, and fissures leading from it in every direction. These fissures, however small they may be, should be followed up, and, *generally*, if a slight ridge interpose between them and other cavities, it should be cut away, and every fissure should be made of sufficient depth and width to retain the gold securely.

One word in regard to complicate fillings in the incisors and cuspids, and I am done, for I would not by exhausting the subject, deprive you of that pleasure, even if I could.

In these teeth, when worn down from occlusion (as is most often the case when grinders have been lost), we may find cavities in the approximal surfaces, which, from the wear of mastication, would, when filled as simple cavities, be soon broken and the fillings dislodged. In cases of this kind, it would be better to cut through the wall at the cutting edge, (or what has really become the grinding surface) and form a groove with under cuts or pits, then, by carrying the foil through this channel from one approximal cavity to the other, build upon or cover the grinding surface as the necessities of the case may require. This is a single operation, and the mode of proceeding and its advantages are obvious, and doubtless familiar to you all.

And now, gentlemen, without supposing for a moment that I have offered a single new idea to this society upon the subject of complicate fillings, I have performed the duty assigned to me by the Executive Committee to the best of my ability.

